# Intra-gastric balloon with lifestyle modification: a promising therapeutic option for overweight and obese patients with metabolic dysfunction-associated steatotic liver disease

**DOI:** 10.1007/s11739-023-03417-2

**Published:** 2023-09-12

**Authors:** A. M. van Dijk, M. de Vries, F. El-Morabit, S. T. Bac, M. W. Mundt, L. E. van der Schuit, M. M. C. Hirdes, M. Kara, J. de Bruijne, S. van Meer, H. A. H. Kaasjager, H. W. de Valk, F. P. Vleggaar, K. J. van Erpecum

**Affiliations:** 1 Department of Dietetics, University Medical Center Utrecht, D01.314, Po Box 85500, Utrecht, 3508 GA The Netherlands; 2 Department of Internal Medicine, University Medical Center Utrecht, Utrecht, The Netherlands; 3 Department of Gastroenterology and Hepatology, University Medical Center Utrecht, Utrecht, The Netherlands; 4 Department of Gastroenterology and Hepatology, Bergman Clinics, Bilthoven, The Netherlands; 5 Present Address: Department of Internal Medicine, Amsterdam University Medical Center, Amsterdam, The Netherlands; 6 Present Address: Flevoziekenhuis, Department of Gastroenterology and Hepatology, Almere, The Netherlands

**Keywords:** Metabolic dysfunction-associated steatotic liver disease, Obese, Intra-gastric balloon, Lifestyle modification

## Abstract

**Background:**

Data on effects of intra-gastric balloon (IGB) on metabolic dysfunction-associated steatotic liver disease (MASLD) are scarce, in part with contradictory results, and mainly obtained in tertiary care patients with diabetes and other comorbidities. We here explore effects of IGB in patients with MASLD referred to a first-line obesity clinic.

**Methods:**

In this prospective cohort study, patients with at least significant fibrosis (≥ F2) and/or severe steatosis (S3) according to screening transient elastography (FibroScan®) were offered a second FibroScan® after 6 months lifestyle modification with or without IGB (based on patient preference). *Results*: 50 of 100 consecutively screened patients (generally non-diabetic) qualified for repeated evaluation and 29 (58%) of those had a second FibroScan®. At baseline, at least significant fibrosis was present in 28% and severe steatosis in 91%. IGB was placed in 19 patients (59%), whereas 10 patients (41%) preferred only lifestyle modification (no differences in baseline characteristics between both groups). After 6 months, liver stiffness decreased markedly in the IGB group (median: from 6.0 to 4.9 kPa, p = 0.005), but not in the lifestyle modification only group (median: from 5.5 to 6.9 kPa, p = 0.477). Steatosis improved in both groups, (controlled attenuation parameter values; IGB, mean ± SD: from 328 ± 34 to 272 ± 62 dB/m, p = 0.006: lifestyle modification only, mean ± SD: from 344 ± 33 to 305 ± 43 dB/m: p = 0.006).

**Conclusion:**

Both steatosis and fibrosis improve markedly in overweight/obese patients with MASLD after 6 months IGB combined with lifestyle modification. Our results warrant further research into long-term effect of IGB in these patients.

**Supplementary Information:**

The online version contains supplementary material available at 10.1007/s11739-023-03417-2.

## Introduction

In a recent multi-stakeholder effort under the auspices of the American Association for Study of Liver Disease (AASLD) and the European Association for Study of the Liver (EASL) in collaboration with the Asociación Latinoamericana para el Estudio del Hígado (ALEH), the terms metabolic dysfunction-associated fatty liver disease (MAFLD) and non-alcoholic steatohepatitis (NASH) were replaced by metabolic dysfunction-associated steatotic liver disease (MASLD) and metabolic dysfunction-associated steatohepatitis (MASH). The incentive for this change was to accurately capture disease etiology and to avoid stigmatizing [1]. We here adopt the new nomenclature. MASLD is the most common liver disease worldwide and a leading cause of liver-related morbidity and mortality, affecting one third of the global population [2–4]. Prevalence of MASLD increases worldwide, due to spread of Western diet and increasing body weight. Weight reduction of at least 5% can lead to improvement of blood liver tests [5] and decreased liver steatosis [6, 7]. Weight reduction of 7–10% may even improve lobular inflammation [7, 8], ballooning injury [7, 8] and fibrosis [7] with potential resolution of metabolic dysfunction-associated steatohepatitis (MASH) and MASLD [6, 9, 10]. Treatment of MASLD should therefore focus on weight reduction of at least 5% and preferably 10% [11, 12].

Effects of energy-restricted diets with or without a complementary exercise program on weight loss and MASLD are generally disappointing [9, 13]. Bariatric surgery is more effective but carries an appreciable risk of postoperative complications and even mortality, especially in patients with preexisting liver disease [14].

In contrast to bariatric surgery, intra-gastric balloon (IGB) placement aims to achieve clinically significant weight loss, with minimal serious adverse events [15–17]. A limited number of studies have indicated that IGB is effective in achieving such weight loss [18–22], with improvement in blood liver tests [18, 19, 21], steatosis [18, 19, 21, 22] as well as fibrosis [21, 22]. These studies are generally characterized by a large number of participants in tertiary care, with type 2 diabetes mellitus (T2DM) and other comorbidities. Good or reasonable accurate methodology for MASLD evaluation such as histology (2 studies), FibroScan® (2 studies) and ultrasound (one study) were used in 5 previous publications (Supplementary Table 1). Number of included patients was often limited, with significant loss of follow up. Whereas hepatic steatosis generally improved, contradictory results were reported for hepatic fibrosis (Supplementary Table 1). Importantly, distinction between three (lean, overweight/obesity- and diabetes-associated MASLD) or even more subgroups of MASLD has recently been suggested, considering different risk of liver fibrosis as well as risk of cardiovascular, cancer-related and all-cause mortality in these subgroups [23–26]. Of note, literature on effects of IGB on liver disease in non-diabetic overweight or obese persons is scarce and results of IGB in this subgroup could differ from diabetic subgroups.

The current non-invasive standard to diagnose liver fibrosis is liver stiffness measurement (LSM) by transient elastography with the aid of FibroScan®, with high diagnostic and prognostic accuracy [27]. Other non-invasive tests like fibrosis-4 (FIB-4) index, NAFLD fibrosis score (NFS) and enhanced liver fibrosis (ELF) test are less expensive, but an appreciable risk of false positive and false negative results limits their usefulness [27].

We therefore investigate in the current work, with the aid of FibroScan® potential beneficial effects of IGB on MASLD in persons referred to a first-line obesity clinic for weight reduction.

## Methods

### Study design and setting

In this prospective cohort study, all adult participants (≥ 18 years) who were referred to a first-line Dutch obesity clinic for potential treatment with an IGB for overweight or obesity (BMI 25–45) in the period September 2018-February 2021 were asked to participate in this study. The local Medical Ethical Committee had no objection to the study (research protocol 15/705).

Inclusion criteria were referred to the Obesity clinic for weight reduction and patient written informed consent. Exclusion criteria were: any contraindication for IGB placement or FibroScan®. Contraindications to IGB placement were BMI < 25 or > 45, pregnancy, intrahepatic mass, prior intestinal surgery, hiatal hernia > 5 cm, gastric lesions with risk of bleeding, any organic disease of the upper gastro-intestinal tract, inflammatory disease of the gastro-intestinal tract, anti-inflammatory drugs, anticoagulants or steroids, current or history of substance abuse. Theoretical contraindications for FibroScan® include: pregnancy, pacemaker, implantable cardioverter defibrillator, and (as detected by standard physical examination) liver congestion, extrahepatic cholestasis, intrahepatic mass or ascites.

### Screening procedure and details of intra-gastric balloon (IGB) placement and lifestyle modification therapy

Psychological difficulties may occur in some patients after IGB placement, such as problems with mood, eating, anxiety and substance abuse [28]. Screening for potential psychopathology before bariatric surgery is therefore advised in authorative guidelines [29]. In the current study, psychopathology screening was done before acceptance for IGB placement with the aid of the SQ-48 questionnaire [30]. Further psychological counseling was offered if indicated. Choice of adding IGB to lifestyle modification or not was based on patient preference. In case of IGB placement, after initial endoscopic screening, the ORBERA™ system was positioned under endoscopic control in the gastric fundus. The IGB was filled with 400–500 ml sterile NaCl 0.9% and 2 ml methylene blue. In the first 24h after IGB placement, only clear liquid was permitted. During the first seven days, a gradual progression from clear liquid to liquid and subsequently to semi-liquid diet was advised. Thereafter, participants proceeded to solid diet. The IGB was removed after 6 months.

Lifestyle modification therapy was prescribed to all participants (with and without IGB placement), consisting of a hypocaloric diet in combination with an increase of physical activity. Caloric needs were calculated by Harris and Benedict formula [31], corrected for physical activity and deducted with 500 kcal. According to the protocol, participants were advised to have moderate-intensity aerobic physical activity for 150 min a week and two times a week a muscle and bone strengthening exercise program [11, 32].

### Data collection and measurements

Blood laboratory tests (alkaline phosphatase (ALP), gamma glutamyl transpeptidase (GGT), alanine aminotransferase (ALT) and glucose) were obtained at baseline and upon IGB removal (after 6 months follow-up). Weight and height were obtained at baseline and after six months. Vibration-controlled transient elastography measurements were performed with the aid of FibroScan® 502 (Echosens) by a trained investigator at baseline and after six months. In order to achieve valid measurements, FibroScan® was performed in the fasting state in supine position with their right arm placed under the head. Either the M- or XL-probe was used. The probe was placed in the intercostal space of the 10^th^ and 12^th^ rib in the midaxillary line. FibroScan® was considered successful if at least 10 valid measurements were obtained and considered reliable if interquartile range from median of liver stiffness measurement (LSM) was ≤ 30%. Hepatic fibrosis was assessed by LSM and hepatic steatosis was assessed by controlled attenuation parameter (CAP). To obtain optimal accuracy in a MASLD population, cutoff values for hepatic fibrosis (LSM) and hepatic steatosis (CAP) were defined as: no/mild fibrosis (F0/F1): < 8.2 kPa; significant fibrosis (F2): ≥ 8.2–9.6 kPa; advanced fibrosis (F3): ≥ 9.7–13.5 kPa or cirrhosis (F4): ≥ 13.6 kPa and no steatosis (S0): < 274 dB/m; mild steatosis (S1): 274–289 dB/m; moderate steatosis (S2): 290–301 dB/m or severe steatosis (S3): ≥ 302 dB/m [33, 34]. Participants with at least significant fibrosis (≥ F2) and/or severe steatosis (S3) were offered a second FibroScan® measurement after 6 months to explore potential additional beneficial effects of IGB placement. The reason for this policy was that we considered improvement or deterioration after 6 months unlikely in case of favorable baseline FibroScan® LSM and CAP measurements.

### Study aims and intended patient inclusion numbers

The primary aim of our study was to explore potential effects of IGB on liver fibrosis in MASLD patients. Based on the two available IGB studies with FibroScan® (26 resp 34 patients included) [21, 22], a power of 80% and a two-sided significance level of 0.05, assuming a response rate of 90% for initial screening FibroScan®, with 50% qualifying for a second FibroScan® after 6 months therapy according to our predefined criteria, 70% of included patients choosing IGB therapy (based on experience of our Obesity clinic) and a drop-out rate between IGB combined with lifestyle modification [22]), approximately 180 patients needed to be invited for screening for our study. However, clinical characteristics of our study patients and consent for FibroScan® could well differ from previous study populations [21, 22] and other assumptions could also not turn out correct. We therefore decided to invite as many patients as possible but at least 200 patients for screening FibroScan®. Secondary aims of our study were effects of IGB on liver steatosis, body weight and BMI, effects of lifestyle modification only on MASLD, weight and BMI and comparison between IGB combined with lifestyle modification versus only lifestyle modification.

### Statistical analysis

Statistical data analyses were carried out using SPSS version 27.0. A two-sided p-value < 0.05 was considered statistically significant. Categorical data are expressed as absolute numbers with percentages. In case of normal distribution, continuous variables are always presented as mean ± SD [range]. In case of non-parametric distribution, data are given as median [range]. Fisher’s exact tests, Mann–Whitney U tests or independent samples t-tests were applied for comparison of two groups as appropriate. Change of patient characteristics between baseline and six months later were evaluated with Wilcoxon signed-rank tests or paired samples t-tests as appropriate.

## Results

### Baseline

**Fig. 1:**
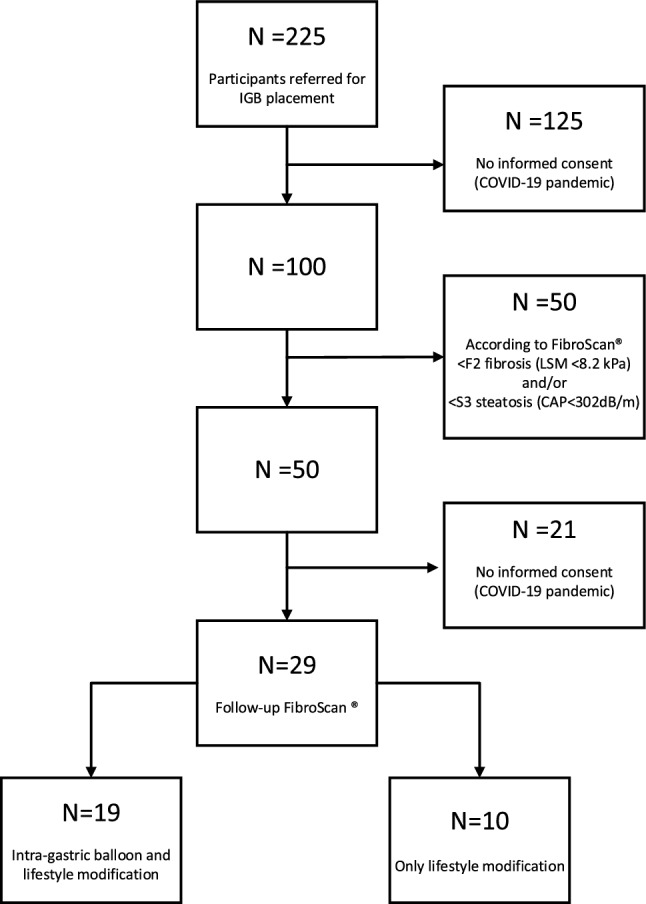
Flowchart of patient inclusion. CAP, controlled attenuation parameter; LSM liver stiffness measurement

Of all 225 persons referred for IGB placement during the study period, 100 participants were screened by FibroScan®. The other 125 persons did not consent to have FibroScan® in addition to lifestyle modification with or without IGB, or such additional FibroScan® for research purposes was not allowed by Hospital regulations during the COVID-19 pandemic (flowchart in Fig. 1). Alcohol abuse, diabetes or hypertension occurred in only a small minority of screened participants, and there was no history of chronic liver disease or cardiovascular events in any case.

The baseline demographic and liver-specific characteristics of the 100 screened participants are given in Supplementary Table 2.

Fifty participants qualified for follow-up FibroScan® based on our pre-defined criteria (i.e. at least significant fibrosis and/or severe steatosis). Male sex and higher BMI were associated with qualifying for follow up FibroScan®. Of the 50 qualifying patients, 29 participants had a second FibroScan® six months after baseline measurement (19 IGB, 10 lifestyle modification alone: Flowchart Fig. 1). During the 6-month period, none of the patients had additional (pharmacological or surgical) treatment apart from the lifestyle modification with or without IGB. Follow-up FibroScan® was not performed in the other 21 participants, mainly because of the COVID-19 pandemic. Clinical characteristics of the 29 participants with follow-up FibroScan® and the 21 participants without follow-up FibroScan® were highly similar (Supplementary Table 3).

### Effects of IGB placement

**Fig. 2:**
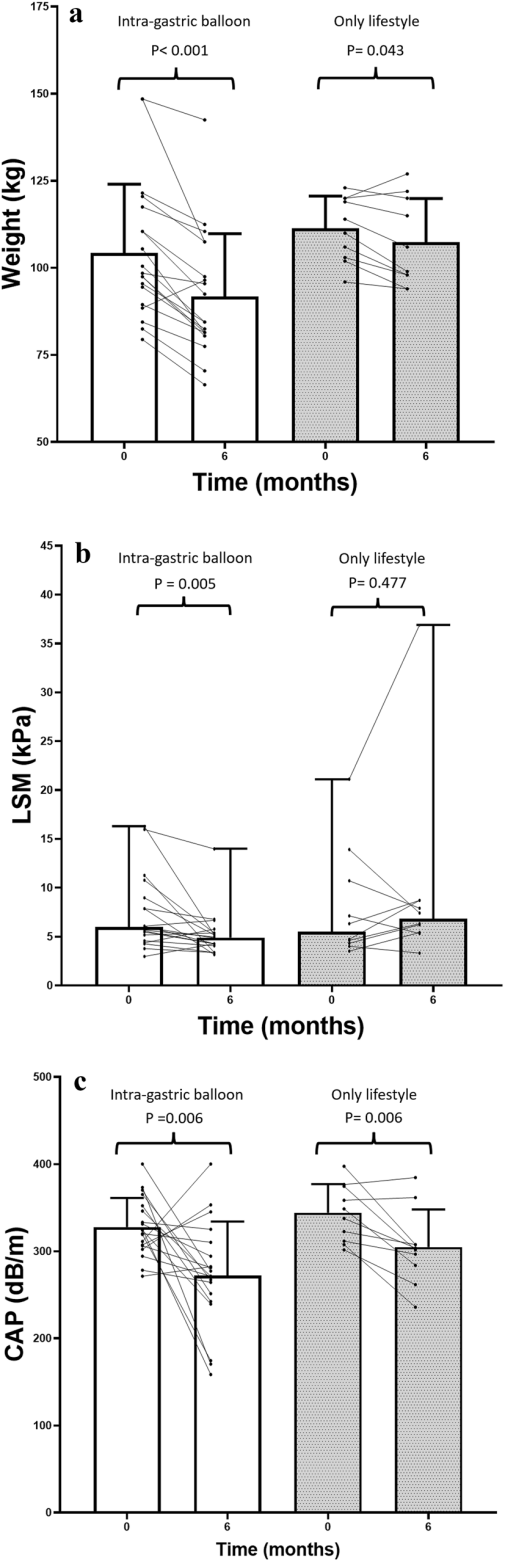
Effects of 6 months intra-gastric balloon with lifestyle modification or lifestyle modification alone on weight (mean ± SD; **a**), liver fibrosis (median [range]; **b**) and steatosis (mean ± SD; **c**)

Significant weight loss and reduction in BMI were achieved after 6 months IGB (mean weight at baseline 104 kg and at follow-up 92 kg, p < 0.001: Fig. 2a; BMI at baseline 34.3 kg/m^2^ and at follow-up 30.2 kg/m^2^, p < 0.001: Table 1). As shown in Fig. 2b and c, clear improvements in LSM and CAP values were also observed after 6 months IGB (median LSM at baseline 6.0 kPa and at follow-up 4.9 kPa, p = 0.005: Fig. 2b; mean CAP at baseline 328 dB/m and at follow-up 272 dB/m, p = 0.006: Fig. 2c). Steatosis grade also improved significantly with IGB (at baseline S0 in 5%, S1 in 5%, S2 in 5% and S3 in 85% and at follow-up S0 in 53%, S1 in 16%, S2 in 5% and S3 in 26%, p < 0.001).

**Table 1:** Changes in anthropometric, FibroScan ® and blood laboratory tests after intra-gastric balloon or lifestyle modification alone

Clinical parameter	Total (N = 29)	IGB (N = 19)			Lifestyle modification (N = 10)			p-value^a^	
		T0	T1	p-value^b^	T0	T1	p-value^b^	T0	T1
Age (years), mean ± SD [range]	46 ± 12 [21–66]	46 ± 12 [22–66]	–	–	47 ± 13 [21–65]	–	–	.867	–
Male gender, n(%)	10(35)	6(32)	–	–	4(40)	–	–	.698	–
Weight (kg), mean ± SD [range]	107 ± 17 [79–148]	104 ± 20 [79–148]	92 ± 18 [66–142]	** < .001**	111 ± 9 [96–123]	107 ± 13 [94–127]	**.043**	.301	**.023**
Weight change (kg), mean ± SD [range]	− 10 ± 9 [− 41—+ 8]	–	− 13 ± 10 [− 41– + 8]	–	–	− 4 ± 5 [− 11– + 7]	–	–	**.016**
More than 5% weight loss during follow-up, n(%)	–	–	16(84)	–	–	4(40)	–	–	**.032**
More than 10% weight loss during follow-up, n(%)	–	–	12(63)	–	–	1(10)	–	–	**.008**
BMI (kg/m^2^), mean ± SD [range]	35.1 ± 3.5 [28.4–42.5]	34.3 ± 3.2 [28.4–40.2]	30.2 ± 3.5 [24.2–38.1]	** < .001**	36.6 ± 3.6 [32.2–42.5]	35.3 ± 4.6 [29.7–45.0]	*.051*	*.087*	**.002**
LSM (kPa), median [range]	6.0 [3.0–21.1]	6.0 [3.0–16.3]	4.9 [3.2–14.0]	**.005**	5.5 [3.5–21.1]	6.9 [3.3–36.9]	.477	.830	**.007**
∆LSM (kPa), median [range]	− 0.9 [− 11.1– + 15.8]	–	− 1.1 [− 11.1– + 1.4]	–	–	1.8 [− 6.5– + 15.8]	–	–	**.038**
Fibrosis stage, n(%) F0/F1 F2 F3 F4	21(72) 1(3) 3(10) 4(15)	14(75) 1(5) 2(10) 2(10)	18(95) 0(0) 0(0) 1(5)	.125	7(70) 0(0) 1(10) 2(20)	7(70) 2(20) 0(0) 1(10)	.625	.878	.105
CAP (dB/m), mean ± SD [range]	333 ± 34 [271–400]	328 ± 34 [271–400]	272 ± 62 [158–400]	**.006**	344 ± 33 [302–398]	305 ± 43 [236–385]	**.006**	.220	.151
∆CAP (dB/m), mean ± SD [range]	− 50 ± 66 [− 203– + 89]	–	− 55 ± 78 [− 203– + 89]	–	–	− 39 ± 34 [− 90– + 8]	–	–	.450
Steatosis grade, n(%) S0 S1 S2 S3	1(3) 1(3) 1(3) 26(91)	1(5) 1(5) 1(5) 16(85)	10(53) 3(16) 1(5) 5(26)	** < .001**	0(0) 0(0) 0(0) 10(100)	2(20) 1(10) 1(10) 6(60)	.125	1.000	.229
GGT (IU/L), median [range]	30.1 [10–130]	29 [10–65]	22 [9–43]	** < .001**	37 [19–130]	–	–	.306	–
GGT upper limit of normal, n(%) ^c^	5(23)	4(22)	2(11)	.625	1(25)	–	–	1.000	–
ALP (IU/L), median [range]	76 [46–185]	80 [63–185]	71 [50–173]	**.003**	53 [46–76]	–	–	**.012**	–
ALP upper limit of normal, n(%) ^c^	1(4)	1(5)	1(5)	1.000	0(0)	–	–	1.000	–
ALAT (IU/L), median [range]	32 [15–82]	32 [15–63]	19 [13–42]	** < .001**	38 [20–82]	–	–	.463	–
ALAT upper limit of normal, n(%)^c^	10(44)	8(42)	2(11)	**.031**	2(50)	–	–	1.000	–
ALAT above 100 IU/L, n(%)	0(0)	0(0)	0(0)	1.000	0(0)	–	–	–	–
Glucose (mmol/L), median [range]	5.3 [4.4–10.9]	5.2 [4.4–10.9]	5.2 [4.2–12.1]	.892	5.4 [5.3–7.2]	–	–	.188	–
Diabetes, n(%)	1(4)	0(0)	–	–	1(11)	–	–	.321	–
Hypertension, n(%)	4(14)	2(11)	–	–	2(22)	–	–	.574	–
Alcohol use, n(%)	13(45)	9(48)	–	–	4(40)	–	–	1.000	–
Alcohol use, n(%) No/sporadic Moderate Abuse	16(55) 9(31) 4(14)	10(52) 6(32) 3(16)	– – –	–	6(60) 3(30) 1(10)	– – –	–	1.000	–
Period of Intra-gastric balloon placement (days), mean ± SD [range]	–	–	186 ± 20 [118–218]	–	–	–	–	–	–

Categorical data were expressed as absolute numbers with percentage and continuous variables were presented as median [range] in case of non-parametric distribution or as mean ± SD [range] in case of normal distribution

*ALAT* alanine aminotransferase, *ALP* alkaline phosphatase, *BMI* body mass index, *CAP* controlled attenuation parameter; F0/1, no/mild fibrosis (< 8.2kPa); F2, significant fibrosis (≥ 8.2–9.7kPa); F3, advanced fibrosis (≥ 9.7–13.5 kPa); F4, cirrhosis ≥ 13.6 kPa); GGT, gamma glutamyl transpeptidase; LSM, liver stiffness measurement; S0, no steatosis (< 274 dB/m); S1, mild steatosis (274–289 dB/m); S2, moderate steatosis (290–301 dB/m); S3 severe steatosis (≥ 302 dB/m); T0, baseline; T1, 6 months follow-up

^a^ Comparison between groups intra-gastric balloon and only lifestyle modification; Parametric data: independent samples t-test; Non-parametric data: Mann–Whitney U-test; Categorical data: Fisher’s Exact test

^b^ Comparison intragroup between baseline and 6 months; Parametric data: paired samples t-test; Non-parametric data: Wilcoxon-signed rank test; Categorical data: Wilcoxon-signed rank test

^c^ GGT upper limit 38.0 IU/L; ALP upper limit 120.0 IU/L; ALAT upper limit 34.0 IU/L

### Effects of lifestyle modification alone

After 6 months lifestyle modification alone, weight was significantly reduced, while BMI decreased at the border of significance (mean weight at baseline 111 kg and at follow-up 107 kg, p = 0.043: Fig. 2a; BMI at baseline 36.6 kg/m^2^ and at follow-up 35.3 kg/m^2^, p = 0.051). No significant change of LSM values (p = 0.477: Fig. 2b) or fibrosis grade (p = 0.625) were observed in participants with lifestyle modification alone. CAP values improved significantly in participants after 6 months lifestyle modification alone (mean CAP at baseline 344 dB/m and at follow-up 305 dB/m, p = 0.006: Fig. 2c).

### Comparison of intra-gastric balloon and lifestyle modification alone

For the entire group, mean weight loss after six months was -10 kg. Participants with an IGB lost significantly more weight than those with lifestyle modification alone (mean: for IGB − 13 kg, for lifestyle modification alone − 4 kg, p = 0.016: Table 1). Weight loss of at least 5 or 10% was more frequently achieved in participants with an IGB placed than in participants with lifestyle modification alone (at least 5% weight loss: 84% in IGB and 40% in lifestyle modification alone, p = 0.032; at least 10% weight loss: 63% in IGB and 10% in lifestyle modification alone, p = 0.008).

During six months, median LSM changed with − 0.9 kPa. LSM of participants with IGB improved, which was not the case for LSM of participants with only lifestyle modification (IGB − 1.1 kPa, lifestyle modification alone + 1.8 kPa (p = 0.038)). During six months follow-up, mean CAP change was − 50 dB/m. Magnitude of decrease was highly similar in IGB and lifestyle modification alone (IGB − 55 dB/m, lifestyle modification alone − 39 dB/m, p = 0.450).

## Discussion

The current study suggests that in patients with obesity subtype of MASLD, combined IGB and lifestyle modification can improve liver fibrosis in addition to steatosis. Five available studies with good or reasonably accurate methodology for MASLD evaluation after IGB placement (such as histology, FibroScan®, or ultrasound) generally indicate improvement of steatosis, but contradictory effects on fibrosis (Supplementary Table 1). Two studies with histological evaluation found no improvement [18, 20] and two previous FibroScan® studies [21, 22] reported improvement of liver fibrosis after IGB therapy. The previous studies are generally characterized by limited patient numbers, a large proportion of participants in tertiary care, with type 2 diabetes mellitus (T2DM) and other comorbidities. In the current study, almost all patients were of the obesity subtype, without T2DM or other comorbidities. It should be mentioned that, also in our study, patient numbers are limited. A lower percentage of invited patients than assumed in the power calculation actually had a baseline FibroScan®, largely related to the COVID-19 pandemic. On the other hand, proportion of qualifying patients who had a second FibroScan® was somewhat higher in the IGB group than in the lifestyle modification only group (19/27, 70% versus 10/23, 43%), which favored inclusion of IGB patients for our primary aim (i.e. to explore potential effects of IGB on liver fibrosis). Also, we asked significantly more patients to participate in this study than requested by the power calculation (225 versus 180). As a result, we were able to show beneficial effects of IGB on liver fibrosis that reached statistical significance. Our findings are in line with the two available previous FibroScan® studies [21, 22]

Disappointing results of lifestyle modification only on weight loss in the current study are in line with many previous studies. The absence of an effect on liver fibrosis can be explained by the fact that the limited weight loss did not reach the 7–10% decrease thought to be necessary for reducing fibrosis. Interestingly, improvement of steatosis occurred in our patients, both with combined IGB and lifestyle modification alone. The latter finding suggests that the modest weight loss due to lifestyle modification alone could have at least some beneficial effects in MASLD. Of note, increased physical activity as part of the lifestyle intervention could also have contributed to improvement of steatosis [35]. Weight reduction of at least 5% is considered a prerequisite to achieve significant health effects. As far as MASLD is concerned, at least 5% weight reduction can lead to improvement of liver steatosis [6, 7]. Weight reduction of at least 7–10% may decrease metabolic dysfunction-associated steatohepatitis (MASH) and fibrosis [6, 9, 10]. We found that weight loss of at least 5% (84% resp. 40%, p = 0.032) or at least 10% (63% resp. 10%, p = 0.008) was more frequently achieved in participants with an IGB placed than in participants with lifestyle modification alone. Our study suggests that IGB in addition to lifestyle modification could be more effective than lifestyle modification alone for improving liver fibrosis. Although our patient numbers are small (especially in the lifestyle modification only group), the only other IGB study on MASLD with a disease-control group included only 18 patients (8 IGB, 10 only lifestyle modification) [20].

Although bariatric surgery is considered the most effective intervention for weight loss, this intervention associated with significant risks, especially in cirrhotic patients [14]. Therefore, bariatric surgery is generally considered especially in selected patients at high risk for (recurrent) cardiovascular events rather than for MASLD as the sole indication. The main goal of IGB placement as an alternative for bariatric surgery is to achieve clinically significant weight loss, with minimal serious adverse events. Possibly, temporary IGB could interrupt a vicious circle, and beneficial short-term effects of IGB on obesity and MASLD could motivate MASLD patients for strict long-term adherence to healthy lifestyle. Also, 6 months IGB could be a bridge to more favourable conditions for definitive bariatric surgery in MASLD patients, considering the increased risks of surgery in severely obese patients with liver disease. As far as alternative pharmacologic therapies are concerned, in recent years, promising short-term results in selected patient groups with MASLD have been reported for pharmacologic therapies of such as 7-ethyl chenodeoxycholic acid (Obeticholic acid®) [36, 37], the pan-PPAR (peroxisome proliferator–activated receptor) agonist Lanifibranor [38] and semaglutide [39]. Nevertheless, these pharmacologic therapies lead to markedly increased health costs and potential side effects. Also, long-term treatment of such pharmacologic therapies could be necessary, with long-term risks and uncertain efficacy. Superior cost–benefit of 6-month IGB compared to pharmacologic therapy would only hold true if beneficial effects persist after IGB removal (no data are available yet on this issue).

Several limitations should be acknowledged regarding our study results. First, patient numbers are limited. Second, mainly due to the COVID-19 pandemic, only 60% of qualifying patients had a second measurement, with potential bias as a result. Nevertheless, there were no differences in baseline characteristics between qualifying patients with or without a second FibroScan®, suggesting that our patient group with a second FibroScan® was representative of the entire group (Supplementary Table 3). Third, only selected patients with at least significant fibrosis (≥ F2) and/or severe steatosis (S3) at baseline were invited for a second FibroScan® measurement 6 months later. The rationale for this policy was, that significant improvement is unlikely in patients with normal or at most minor increases of liver stiffness and CAP values at baseline. We cannot exclude that worsening of fibrosis and/or steatosis could have occurred in some patients not qualifying for a second FibroScan®. However, we think that this is improbable in the IGB patients (the group relevant for our primary aim: effect of IGB on fibrosis), considering large weight loss in the 6 months after IGB placement in almost all patients. In the patients who had only lifestyle advices, deterioration of fibrosis and steatosis is more likely, because of their limited weight loss. Therefore, the fact that we did not perform a second FibroScan® in patients with minor or absent fibrosis and steatosis at baseline could in theory have obscured differences between groups with and without IGB (one of our secondary aims), with potential type 2 error. Fourth, FibroScan® may overestimate severity of fibrosis, especially in case of ALT levels > 100 IU/L. No patient in the current study had an ALT level > 100 IU/L (generally accepted cut off value for potential overestimation of fibrosis by FibroScan®). This indicates that the estimation of fibrosis by FibroScan® is not an important limitation in the current study. Fifth, although FibroScan® is considered a reliable non-invasive tool to estimate fibrosis and steatosis, liver histology (the gold standard) was not available. Last, it was impossible because of strict legal privacy regulations, to obtain longer follow-up data than at 6 months (the time point of IGB removal).

In conclusion, our study suggests that combined 6-months intra-gastric balloon and lifestyle modification could be an effective treatment for obese subjects with MASLD/obesity subtype. These findings need to be confirmed in larger studies with long-term follow up after removal of the intra-gastric balloon.

### Supplementary Information

Below is the link to the electronic supplementary material.

Supplementary file1 (DOCX 80 KB)

## Data Availability

The dataset generated during and/or analysed during the current study are not publicly available due to privacy regulation but are available on reasonable request.

## Abbreviations

BMI: Body Mass Index
CAP: Controlled attenuation parameter
LSM: Liver stiffness measurement
MASH: Metabolic dysfunction-associated steatohepatitis
MASLD: Metabolic dysfunction-associated steatotic liver disease
MAFLD: Metabolic dysfunction-associated fatty liver disease
NASH: Non-alcoholic steatohepatitis
pan-PPAR: Peroxisome proliferator–activated receptor) agonist
