# Supplementary material for: Intra-gastric balloon with lifestyle modification: a promising therapeutic option for overweight and obese patients with metabolic dysfunction-associated steatotic liver disease
Source: Intern Emerg Med. 2023 Sep 12;18(8):2271–80. doi: 10.1007/s11739-023-03417-2 (PMC10635963; doi:10.1007/s11739-023-03417-2)
Supplement: Supplementary file 1 — Supplementary file1 (DOCX 80 KB) [file 11739_2023_3417_MOESM1_ESM.docx]

**Supplementary data:**

**Intra-gastric balloon with lifestyle modification: a promising therapeutic option for overweight and obese patients with metabolic dysfunction-associated steatotic liver disease.**

A.M. van Dijk^✉^, M. de Vries, F. El-Morabit, S.T. Bac, M.W. Mundt, L.E. van der Schuit, M.M.C. Hirdes, M. Kara, J. de Bruijne, S. van Meer, H.A.H. Kaasjager, H. W. de Valk, F.P. Vleggaar, K.J. van Erpecum

**Corresponding author**

Anne M. van Dijk, University Medical Center Utrecht, Department of Dietetics, Utrecht, The Netherlands, Email: [adijk20@umcutrecht.nl](about:blank)

| **Supplementary table 1** Literature data on effectiveness of intra-gastric balloon on change in weight, liver function, steatosis and fibrosis. | | | | | | | | |
| --- | --- | --- | --- | --- | --- | --- | --- | --- |
| Author (year), country | Design (period) | Clinical characteristics | | | Intervention | Outcome parameter | Intervention result |  |
|  |  |  | Intervention | Control |  |  |  |  |
| Lee *et al* (2012), Singapore [20] | RCT (2006-2008) | N | 8 | 10 | Intervention:  BioEnterics intra-gastric balloon  6 months  Hypocaloric diet (1200-1500 kcal/day)  Walk/jog 30 min daily  Control:  Hypocaloric diet (1200-1500 kcal/day)  Walk/jog 30 min daily | NAFLD activity score  Steatosis score (hist.)  Fibrosis score (hist.)  Aminotransaminases  BMI | Improvement in both, but more in intervention  Improvement in both  No effect in both  No effect in both  Improvement in both, but more in intervention |  |
|  |  | Age | 43 [20] | 47 [15] |  |  |  |  |
|  |  | Male (%) | 3 [38] | 8 [80] |  |  |  |  |
|  |  | BMI | 30.3 [4.2] | 32.4 [6.7] |  |  |  |  |
|  |  | Steatosis score | 2.0 [1.0] | 2.0 [1.3] |  |  |  |  |
|  |  | Fibrosis score | 1.0 [0.8] | 1.0 [1.0] |  |  |  |  |
|  |  | DM (%) | 1 (13) | 1 (10) |  |  |  |  |
|  |  | Pop. | Obese  Histologic evidence of NASH |  |  |  |  |  |
|  |  |  |  |  |  |  |  |  |
| Forlano *et al* (2019), Italy [19] | Prospective (January 2004- July 2007) | N | 120 | - | Intervention:  BioEnterics intra-gastric balloon  6 months  Hypocaloric diet (1000-1200 kcal/day) | Steatosis (US)  ALAT  GGT  Weight/BMI | Improvement  Improvement  Improvement  Improvement |  |
|  |  | Age | 38.6 ± 12 | - |  |  |  |  |
|  |  | Male (%) | 43 (36) | - |  |  |  |  |
|  |  | Weight (kg) | 118.8 ± 2.4 [77-188] | - |  |  |  |  |
|  |  | BMI | 43.1 ± 8 [30-67] | - |  |  |  |  |
|  |  | S 0-2  S 3 | 68 (57)  52 (43) | - |  |  |  |  |
|  |  | DM (%) | 16 (13) | - |  |  |  |  |
|  |  | HT | 36 (30) | - |  |  |  |  |
|  |  | MI | 2.5% | - |  |  |  |  |
|  |  | Pop. | Obese | - |  |  |  |  |
|  |  |  |  |  |  |  |  |  |
| Bazerbachi *et al*. (2021), USA [18] | Prospective (October 2016-March 2018) | N | 21 | - | Intervention:  Orbera ®  6 months  Hypocaloric diet (1200-1500 kcal/day)  Cardiovascular activity 5x/w 30 min | NAFLD activity score  Steatosis (hist.)  Fibrosis stage (hist.)  Aminotransaminases  Liver stiffness (MRE)  Weight/BMI | Improvement  Improvement  n.s.  Improvement  Improvement  Improvement |  |
|  |  | Age | 54 [34-65] | - |  |  |  |  |
|  |  | Male (%) | 4 (9) | - |  |  |  |  |
|  |  | Weight (kg) | n.a. | - |  |  |  |  |
|  |  | BMI | 44 [32-55] | - |  |  |  |  |
|  |  | S 0  S 1  S 2 | 1 (5)  14(67)  6 (28) | -  -  - |  |  |  |  |
|  |  | F1A  F1B  F1C  F2  F3 | 6 (29)  1 (5)  2 (10)  7 (32)  5 (24) | -  -  -  -  - |  |  |  |  |
|  |  | DM (%) | 11 (52) | - |  |  |  |  |
|  |  | HT | 57% | - |  |  |  |  |
|  |  | Pop. | Obese  Radiological proven hepatic steatosis and early fibrosis.  Noncirrhotic. | - |  |  |  |  |
|  |  |  |  |  |  |  |  |  |
| Salomone *et al* (2021), Italy [21] | Retrospective (January 2019- June 2020) | N | 26 | - | Orbera ® intra-gastric balloon  6 months  Hypocaloric diet (1000-1300 kcal/day; 100 kcal deficit)  Aerobic activity (3x/w 30 min) | LSM (TE)  Fibrosis stage (TE)  CAP (TE)  Steatosis stage (TE)  Aminotransaminases  GGT  Weight | Improvement  Improvement  Improvement  Improvement  Improvement  Improvement  Improvement |  |
|  |  | Age | 53 [44-62] | - |  |  |  |  |
|  |  | Male (%) | 69% | - |  |  |  |  |
|  |  | Weight (kg) | 106 ± 19.7 | - |  |  |  |  |
|  |  | BMI | 35.1 ± 4.7 | - |  |  |  |  |
|  |  | CAP (dB/m) | 355 [298-400] | - |  |  |  |  |
|  |  | LSM (kPa) | 13.3 ± 3.2 | - |  |  |  |  |
|  |  | DM (%) | 10 (38) | - |  |  |  |  |
|  |  | HT | 65% | - |  |  |  |  |
|  |  | Pop. | Obese with NAFLD and clinically fibrosis | - |  |  |  |  |
|  |  |  |  |  |  |  |  |  |
| Vijayaraghavan *et al* (2022), India [22] | Prospective (2017-2020) | N | 56* | - | Spatz3^TM^  6 months  Hypocaloric diet (1000 kcal/day or at least 500-800 less than estimated) | LSM (TE)  CAP (TE)  Aminotransaminase  Weight/BMI | Improvement  Improvement  No effect  Improvement |  |
|  |  | Age | 58.3 ± 10.33 | - |  |  |  |  |
|  |  | Male (%) | 73 | - |  |  |  |  |
|  |  | Weight (kg) | 96.5±15.0 | - |  |  |  |  |
|  |  | BMI | 35.2±3.9 | - |  |  |  |  |
|  |  | CAP (dB/m) | 310.8±50.7 | - |  |  |  |  |
|  |  | LSM (kPa) | 27.4 ± 14.6 | - |  |  |  |  |
|  |  | DM (%) | 39 (70) | - |  |  |  |  |
|  |  | HT | 11 (10) | - |  |  |  |  |
|  |  | Pop. | Obese  NASH  Chronic liver disease/cirrhosis | - |  |  |  |  |
| ALAT, alanine aminotransferase; BMI, body mass index; CAP, controlled attenuation parameter; DM, diabetes mellitus; F, fibrosis; GGT, gamma glutamyl transpeptidase; Hist., histologic; HT, hypertension; LSM, liver stiffness measurement; MI, myocardial infarct; MRE, magnetic resonance elastography; NAFLD, non-alcoholic fatty liver disease; NASH, nonalcoholic steatohepatitis; Pop, population; RCT, randomized controlled trial; S, steatosis; TE, transient elastography; US, ultrasound.  Categorical data were expressed as absolute numbers with percentage and continuous variables were presented as median [range] in case of non-parametric distribution or as mean ± SD [range] in case of normal distribution. *34 with second FibroScan®.  One study was not presented because results of IGB and gastric banding were not separated,[39] another study was not presented because they focused on decompensated liver cirrhosis [40]and one other was not presented because no clear overall results were presented. [41] Studies solely focusing on liver blood laboratory tests [42, 43] or liver volume[44–46] and not on fibrosis/steatosis are not presented. | | | | | | | |  |

| Supplementary Table 2 Baseline characteristics in participants screened with FibroScan® before combined intra-gastric balloon placement and lifestyle modification or lifestyle modification alone | | | | |
| --- | --- | --- | --- | --- |
| Clinical parameter | Total  (N=100) | Intra-gastric balloon  (N=59) | Lifestyle modification  (N=41) | p-value ^a^ |
| Age (years), mean±SD [range] | 45±10  [21-66] | 45±10  [22-66] | 46±10  [21-65] | .684 |
| Male gender, n(%) | 26(26) | 14(24) | 12(29) | .644 |
| Weight (kg), mean±SD [range] | 101±15  [74-148] | 100±15  [76-148] | 102±14  [74-139] | .398 |
| BMI (kg/m^2^), mean±SD [range] | 33.3±3.4  [25.6-43.0] | 32.9±3.1  [27.6-40.2] | 33.9±3.7  [25.6-43.0] | .151 |
| LSM (kPa), median [range] | 4.7 [1.9-35.0] | 4.7 [2.4-16.3] | 4.6 [1.9-35.0] | .987 |
| Fibrosis stage, n(%)  F0/F1  F2  F3  F4 | 86(86)  3(3)  6(6)  5(5) | 52(88)  1(2)  4(7)  2(3) | 34(83)  2(5)  2(5)  3(7) | .665 |
| F3 or F4 fibrosis, n(%)^b^ | 11(11) | 6(10) | 5(12) | .472 |
| CAP (dB/m), mean±SD [range] | 292±62  [100-400] | 284±60  [100-400] | 303±64  [100-400] | .133 |
| Steatosis grade, n(%)  S0  S1  S2  S3 | 34(34)  8(8)  12(12)  46(46) | 23(39)  4(7)  8(13)  24(41) | 11(26)  4(10)  4(10)  22(54) | .472 |
| GGT (IU/L), median [range] | 26 [7-130] | 23 [7-75] | 35 [12-130] | .176 |
| GGT upper limit of normal, n(%)^b^ | 19(26) | 14(25) | 5(31) | .748 |
| ALP (IU/L), median [range] | 74 [24-185] | 75 [24-185] | 74 [43-117] | .325 |
| ALP upper limit of normal, n(%)^b^ | 4(5) | 4(7) | 0(0) | .567 |
| ALAT (IU/L), median [range] | 25 [13-106] | 24 [13-98] | 28 [18-106] | .496 |
| ALAT upper limit of normal, n(%)^b^ | 24(30) | 17(29) | 7(35) | .588 |
| ALAT > 100 IU/L, n(%) | 1(1) | 0(0) | 1(5) | .253 |
| Glucose (mmol/L), median [range] | 5.2 [4.1-13.0] | 5.2 [4.1-12.7] | 5.3 [4.2-13.0] | .684 |
| Diabetes, n(%) | 1(1) | 0(0) | 1(3) | .402 |
| Hypertension, n(%) | 9(9) | 5(9) | 4(11) | .734 |
| Smoking state, (n%)^c^  Never smoked  Previously smoked  Current smoker | 19 (33)  31 (53)  8 (14) | 9 (26)  20 (57)  6 (17) | 10 (44)  11 (48)  2 (9) | 0.373 |
| Alcohol use, n(%) | 49(49) | 31(53) | 18(44) | .423 |
| Alcohol use, n(%)  No/sporadic  Moderate  Abuse | 51(51)  37(37)  12(12) | 28(47)  23(39)  8(14) | 23(56)  14(34)  4(10) | .683 |
| Period of Intra-gastric balloon placement (days), mean±SD [range] |  | 179±41  [12-245] |  |  |
| Categorical data were expressed as absolute numbers with percentage and continuous variables were presented as median [range] in case of non-parametric distribution or as mean ± SD [range] in case of normal distribution.  Abbreviations: ALAT, alanine aminotransferase; ALP, alkaline phosphatase; BMI, body mass index; CAP, controlled attenuation parameter; F0/1, no/mild fibrosis (<8.2kPa); F2, significant fibrosis (≥8.2-9.7kPa); F3, advanced fibrosis (≥9.7-13.5 kPa); F4, cirrhosis ≥13.6 kPa); GGT, gamma glutamyl transpeptidase; LSM, liver stiffness measurement; S0, no steatosis (<274 dB/m); S1, mild steatosis (274-289 dB/m); S2, moderate steatosis (290-301 dB/m); S3 severe steatosis (≥302 dB/m).  ^a^ Parametric data: independent samples t-test; Non-parametric data: Mann-Whitney U-test; Categorical data: Fisher’s Exact test.  ^b^ GGT upper limit 38.0 IU/L; ALP upper limit 120.0 IU/L; ALAT upper limit 34.0 IU/L.  ^c^ Available for 58 patients | | | | |

| **Supplementary table 3** Clinical characteristics at baseline and 6 month-follow up in participants eligible for second FibroScan® after 6 months (i.e. severe steatosis (S3) and/or fibrosis (≥F2) according to FibroScan® at baseline) do not differ between subgroups with or without a second FibroScan®. | | | | | | | | | |
| --- | --- | --- | --- | --- | --- | --- | --- | --- | --- |
| Clinical parameter | Total  (N=50) | Second FibroScan® performed  (N=29) | | | Second FibroScan® not performed  (N=21) | | | p-value ^a^ | |
|  |  | T0 | T1 | p-value^b^ | T0 | T1 | p-value^b^ | T0 | T1 |
| Age (years), mean±SD [range] | 47±10  [21-66] | 46±12  [21-66] | - | - | 47±8 [31-61] | - | - | .690 | - |
| Male gender, n(%) | 18(36) | 10(35) | - | - | 8(38) | - | **-** | 1.000 | - |
| Weight (kg), mean±SD [range] | 105±16  [76-148] | 107±17 [79-148] | 97±18  [66-142] | **<.001** | 104±16 [76-139] | 98±15 [71-114] | **0.008** | .528 | .944 |
| Weight change (kg), mean ± SD [range] | -9±9  [-41-+8] | - | -10±9  [-41-+8] | - | - | -7±5  [-14-0] | - | - | .439 |
| More than 5% weight loss during follow-up, n(%) | 25(69) | - | 20(69) | - | - | 5(71) | - | - | 1.000 |
| More than 10% weight loss during follow-up, n(%) | 15(42) | - | 13(45) | - | - | 2(29) | - | - | .674 |
| BMI (kg/m^2^), mean±SD [range] | 34.1±3.4  [28.3-42.5] | 35.1±3.5 [28.4-42.5] | 32.0±4.5 [24-45] | **<.001** | 32.8±2.8 [28.3-38.8] | 31±3.0 [26.4-35.3] | **.008** | **.017** | 0.572 |
| LSM (kPa), median [range] | 6.0  [3.0-35] | 6.0  [3.0-21.1] | 5.3  [3.2-36.90] | .151 | 6.0  [3.0-35.0] | - | - | .479 | - |
| Fibrosis stage, n(%)  F0/F1  F2  F3  F4 | 36(72)  3(6)  6(12)  5(10) | 21(72)  1(3)  3(10)  4(15) | 25(86)  2(7)  -  2(7) | *.055* | 15(71)  2(10)  3(14)  1(5) | - | - | .647 | - |
| CAP (dB/m), mean±SD [range] | 339±32  [271-400] | 333±34 [271-400] | 284±57  [158-400] | **<.001** | 346±29 [278-400] | - | - | .175 | - |
| Steatosis grade, n(%)  S0  S1  S2  S3 | 1(2)  2(4)  1(2)  46(92) | 1(3)  1(3)  1(3)  26(91) | 12(41)  4(14)  2(7)  11(38) | **<.001** | 0(0)  1(5)  0(0)  20(95) | - | - | 1.000 | - |
| GGT (IU/L), median [range] | 33  [10-130] | 31  [10-130] | 22  [9-43] | **<.001** | 35  [17-87] | 28  [9-411] | .406 | .359 | .524 |
| GGT upper limit of normal, n (%)^c^ | 12(32) | 5(23) | 2(11) | .625 | 7(44) | 2(33) | 1.000 | .289 | .234 |
| ALP (IU/L), median [range] | 75  [24-185] | 76  [46-185] | 71  [50-173] | **.003** | 75  [24-143] | 75  [37-140] | .688 | .972 | .877 |
| ALP upper limit of normal, n (%)^c^ | 2(5) | 1(4) | 1(5) | 1.000 | 1(6) | 1(17) | 1.000 | 1.000 | .430 |
| ALAT (IU/L), median [range] | 28  [15-98] | 32  [15-82] | 19  [13-42] | **<.001** | 28  [18-98] | 28  [16-81] | 1.000 | .794 | .177 |
| ALAT upper limit of normal, n(%)^c^ | 16(41) | 10(44) | 2(11) | **.031** | 6(38) | 2(33) | 1.000 | .752 | .234 |
| ALAT above 100 IU/L, n(%) | 0(0) | 0(0) | 0(0) | 1.000 | 0(0) | 0(0) | 1.000 | - | - |
| Glucose (mmol/L), median [range] | 5.2  [4.4-13.0] | 5.3  [4.4-10.9] | 5.2  [4.2-12.1] | .892 | 5.2  [4.5-13.0] | 5.2  [4.8-12.1] | .875 | .924 | .462 |
| Diabetes, n(%) | 1(2) | 1(4) |  | - | 0(0) | - | - | 1.000 | - |
| Hypertension, n(%) | 6(13) | 4(14) | - | - | 2(10) | - | - | 1.000 | - |
| Smoking state, (n%)^d^  Never smoked  Previously smoked  Current smoker | 7(28)  15(60)  3(12) | 3 (30)  7 (70)  0 (0) | -  -  - | -  -  - | 4(27)  8(53)  3(20) | -  -  - | -  -  - | 0.426 | - |
| Alcohol use, n(%) | 24(48) | 13(45) | - | - | 11(52) | - | - | .775 | - |
| Alcohol use, n(%)  No/sporadic  Moderate  Abuse | 26(52)  14 (28)  10(20) | 16(55)  9(31)  4(14) | -  -  - | - | 10(48)  5(23)  6(29) | -  -  - | - | .480 | - |
| Intra-gastric balloon, n(%) | 27(54) | 19 (66) | - | - | - | 8 (38) | - | *.085* | - |
| Period of Intra-gastric balloon placement (days), mean±SD [range] | 186±24  [107-218] | - | 186±20 [118-218] | - | - | - | 186±34 [107-212] | - | .953 |
| Categorical data were expressed as absolute numbers with percentage and continuous variables were presented as median [range] in case of non-parametric distribution or as mean ± SD [range] in case of normal distribution.  Abbreviations:  ALAT, alanine aminotransferase; ALP, alkaline phosphatase; BMI, body mass index; CAP, controlled attenuation parameter; F0/1, no/mild fibrosis (<8.2kPa); F2, significant fibrosis (≥8.2-9.7kPa); F3, advanced fibrosis (≥9.7-13.5 kPa); F4, cirrhosis ≥13.6 kPa); GGT, gamma glutamyl transpeptidase; LSM, liver stiffness measurement; S0, no steatosis (<274 dB/m); S1, mild steatosis (274-289 dB/m); S2, moderate steatosis (290-301 dB/m); S3 severe steatosis (≥302 dB/m); T0, baseline; T1, 6 months follow-up .  ^a^ Comparison between groups with and without second FibroScan ® performed; Parametric data: independent samples t-test; Non-parametric data: Mann-Whitney U-test; Categorical data: Fisher’s Exact test.  ^b^ Comparison intragroup between baseline and 6 months; Parametric data: paired samples t-test; Non-parametric data: Wilcoxon-signed rank test; Categorical data: Wilcoxon-signed rank test.  ^c^ GGT upper limit 38.0 IU/L; ALP upper limit 120.0 IU/L; ALAT upper limit 34.0 IU/L.  ^d^ Available in 25 patients. | | | | | | | | | |
